# Tocotrienol-Rich Fraction (TRF) Suppresses the Growth of Human Colon Cancer Xenografts in Balb/C Nude Mice by the Wnt Pathway

**DOI:** 10.1371/journal.pone.0122175

**Published:** 2015-03-25

**Authors:** Jing-Shu Zhang, Shu-Jing Zhang, Qian Li, Ying-Hua Liu, Ning He, Jing Zhang, Peng-Hui Zhou, Min Li, Tong Guan, Jia-Ren Liu

**Affiliations:** 1 Department of Toxicology, Tianjin Centers for Disease Control and Prevention, Tianjin, People’s Republic of China; 2 College of Public Health, Tianjin Medical University, Tianjin, People’s Republic of China; 3 Boston Children’s Hospital and Harvard Medical School, Boston, Massachusetts, United States of America; University of Hong Kong, HONG KONG

## Abstract

Tocotrienols have been shown many biologic functions such as antioxidant, anti-cancer, maintaining fertility and regulating the immune system and so on. In this study, after feeding with tocotrienol-rich fraction from palm oil (TRF) for 2 weeks, Balb/c nude mice were inoculated human colon SW620 cancer cell and then continued to feed TRF for 4 weeks. At termination of experiments, xenografts were removed and determined the expression of Wnt-pathways related protein by immunohistochemistry or western blotting. Liver tissues were homogenated for determining the levels of antioxidative enzymes activity or malondialdehyde (MDA). The results showed that TRF significantly inhibited the growth of xenografts in nude mice. TRF also affected the activity of antioxidative enzymes in the liver tissue of mice. These changes were partly contributed to activation of wnt pathways or affecting their related protein. Thus, these finding suggested that the potent anticancer effect of TRF is associated with the regulation of Wnt signal pathways.

## Introduction

The colon cancer is one of the most common malignancies and threatens human health in the world. According to available references, the vast majority of colon cancer is accompanied by the activation of Wnt signal pathway[1–3]. Wnt signal pathway is a complicated protein interaction network, which has found in the progress of embryonic development and the cancer. Simultaneously, it has participant in the normal physiological processes of adults[3]. Any changes in the composition of Wnt signal pathway will lead to the abnormal of signal transduction, in turn to the cell malignant transformation, and finally, sparking the occurrence of malignant tumors. Wnt signal pathway mainly consists of a series of proteins such as extracellular factor (Wnt), transmembrane receptors Frizzled (Frz), β-catenin and T cell factor (TCF) etc[4]. Activated Wnt signal interactions with the cytoplasmic protein, β-catenin can stay stability and accumulation in the cytoplasm. The cumulated β-catenin then enters the nucleus cooperationg with the TCF to activate the transcription of target genes such as cyclin Dl and c-myc, etc.[4]. Thus, it is noteworthy that different stages in the Wnt signal pathways have different regulatory factors[1, 5].

The active ingredients from natural plants being the first choice possess the characteristic of killing cancer cells and no harm on normal cells. Tocotrienols, as an active ingredient extracted from the grain and palm trees, have a potential potency to inhibit the tumor cell growth [1, 2, 6–9]. In the tocotrienol-rich fraction from palm oil (TRF), 23.5% of them are total tocotrienols which is 10.7% for γ-tocotrienol. In a previous study, TRF could induce a p53-independent death pathway in human colon cancer RKO cells [10]. Eitsuka et al, have reported that the vitamin E which containing high concentrations of tocotrienol (δ-tocotrienol) can inhibit the growth of human colon adenocarcinoma cells by down-regulation the telomerase activity [11]. Xu W et al. observed the function of γ-tocotrienol on human colon cancer HT-29 cells to find that γ-tocotrienol suppressed an important component in Wnt signal pathways—the β-catenin/Tcf in HT-29 cells[12]. In addition, tocotrienols or TRF have been reported several kinds of malignant carcinoma cells *in vitro* and *in vivo*[1, 8, 9, 13–15]. γ-Tocotrienol could inhibit the growth of human gastric cancer cells and chemosensitize it to capecitabine in a xenograft mouse model through the modulation of NF-κB pathway[15]. TRF suppressed the growth of tumor cell by reducing revascularization[13] and tumor gene expression in breast cancer MCF-7 cells inoculation to the nude mice [14]. When compared to the control mice, the expression of the interferon-inducible transmembrane protein-1 gene and the CD59 glycoprotein precursor gene was obviously up-regulated in the TRF-treated mice.

Our previous studies have demonstrated that δ-tocotrienol could induce a paraptosis-like death in colon cancer SW620 cells, and have effects of Wnt signal pathways[2]. Thus, we hypothesized that the similar inhibitory function of tocotrienol might be existent in an animal study. In this study, we were to determine whether TRF inhibited the growth of human colon cancer cells in Balb/c nude mice and how TRF affected the Wnt signal pathways. These results would help us to understand the mechanism of tocotrienols on the anticancer property.

## Materials and Methods

### Ethics Statement

The animal experimental protocol was approved by the Committee on the Ethics of Animal Experiments of Tianjin Center for Disease Control and Prevention (permit number: TJCDC0111). This study was carried out in strict accordance with The People's Republic of China Laboratory Animal Regulations.

### Chemicals and reagents

Reagents for determining hemoglobin, red blood cells, white blood cell reagents, lymphatic, monocytes, neutrophils, eosinophils and basophilic were purchased from Sysmex. The oxidation index detection kits were from NanJing JianCheng Bioengineering Institute (NanJing, China). The PRO-PREP protein extraction solution was from BeiJing SBS Genetech Co., Ltd (BeiJing, China).

### Cell culture

Human colon carcinoma SW620 cells were bought from the Cancer Institute of Chinese Academy of Medical Science (BeiJing, China). The cells were cultured in L-15 medium containing 2 mmol/L L-glutamine and 2 g/L sodium bicarbonate (Gibco), supplemented with 10% fetal bovine serum (FBS, Gibco), antibiotics (100 U/mL of penicillin and 100 μg/mL of streptomycin) at 37°C in a humidified atmosphere with 95% air and 5% CO_2_. Before 90% confluence, cells were passaged using 0.25% trypsin containing 0.02% EDTA.

### Animal treatment

Fifty male (17–22g) and fifty female (14–19g) BALB/c nude mice (6–8 weeks old) were bought from Vital River Laboratory Animal Technology Co., Ltd (Beijing, China). Mice were kept to adapt to the conditions for 5 days before starting the experiment. Animal room temperature is about 20–25°C, relative humidity is about 40–70%. Mice drinking water, bedding and feeding cages have been autoclaved before given to mice.

Mice were randomly divided into 5 groups (20 per group) by body weight, equally in male and female, as following groups: negative control group, tumor model group, and three doses of tocotrienol-rich fraction (TRF, Palm Nutraceuticals Sdn. Bhd., Malaysian) from palm oil groups. The ingredients in TRF are shown in Table 1. TRF was dissolved in soybean oil. Three doses of 5, 10 and 20 mg/kg body weight (b.w.) of TRF which were equivalent to γ-tocotrienol were given the mice by gavage. At the same time, gavage the negative control group and tumor model group with the equal solvent soybean oil. Two weeks later, mice including tumor model group were injected 0.1 mL the logarithmic phase of SW620 cells (1.0×10^7^/mL) into the axilla of each mouse. All animals were observed daily and still were given TRF/soybean oil for 4 weeks. During the experiment, animals free got food and water. After whole blood was collected from eye canthus in each animal under general anesthesia, all animals were euthanized by cervical dislocation. The weight of organs including liver, spleen and kidney, and tumor tissue were measured. Parts of tumor tissues were fixed in the 4% formalin, subsequently dehydrated and embedded in the paraffin for immunohistochemical staining. The rest tumor tissues were frozen in −80°C for western blotting.

**Table 1:** The content of tocotrienols and tocopherol in TRF.

Isomers of Vitamin E	% of TRF	% of total Vitamin E
α-tocopherol	0.1	0.4
α-tocotrienol	2.3	9.8
β-tocotrienol	1.0	4.1
γ-tocotrienol	10.7	45.6
δ-tocotrienol	9.4	40.1
Total	23.5	100.0

TRF: Tocotrienol-rich fraction from palm oil. The data came from Palm Nutraceuticals Sdn. Bhd, Malaysia

### Determination of oxidation index in liver tissues

The liver tissues were homogenated under the ice bath. Oxidation indexes including superoxide dismutase (SOD), glutathione-Px (GSH-Px), malondialdehyde (MDA) and catalase (CAT) were measured in the liver homogenate as described a previous study [16] or followed manufacture’s instruction.

### Immunohistochemistry

The slides of tumor tissues were deparaffinized in xylene and rehydrated through graded alcohol following by immunohistochemistrical staining as described a previous study[17]. Briefly, the sections were heated for 10 min at 95−100°C in 10 mmol/L sodium citrate buffer (pH 6.0). Three percent of hydrogen peroxide was added to the sections to inactivate endogenous peroxidases and then 10% normal goat serum were added to block nonspecific binding. The sections were subsequently incubated at 4°C overnight with anti-Wnt-1, or anti-c-myc, or anti-b-catenin antibodies (Santa Cruz Biotechnology, Inc. Santa Cruz, CA). The sections then were incubated with biotinylated anti-mouse IgG or anti-rabbit IgG (ZSGB-BIO Co., Ltd. BeiJing, China) for 30 min, followed by peroxidase-conjugated streptavidin (ZSGB-BIO Co., Ltd.) for 30 min. The sections were stained with 3, 3′-diaminobenzidine (DAB) for 3 min and counterstained with hematoxylin. The negative control with the omission of the primary antibody also followed the same protocol. The number of positive cells was counted under microscopy (200×). The ratio of positive cells was calculated in each group.

### Western blotting

Total protein extraction from tumor tissues using RIPA buffer (Cell Signaling Technology, Inc. Danvers, MA) and western blotting were performed as described previously studies [18, 19]. The following primary antibodies were used: anti-Wnt-1, anti-β-catenin, anti-c-myc, anti-cyclin D1, anti-MMP-7, anti-axin-2, anti-APC, anti-c-jun, or anti-β-actin antibodies (Santa Cruz Biotechnology, Inc.). The secondary antibodies were corresponding to the type of primary antibody using anti-mouse or anti-rabbit IgG horseradish peroxidase (HRP)-conjugate (1:1,000 dilution, Cell Signaling Technology, Inc.). Protein bands were visualized by the Enhanced Chemiluminescence kit. The quantification of protein bands were measured by FluorChem Imaging Systems (Alpha Innotech). The data were standardized by β-actin.

### Statistical analysis

All data are expressed as means±standard deviation (S.D.). The differences between the control and treated groups were assessed by one-way ANOVA followed by Student-Newman-Keuls (SNK) test. Differences were considered significant at *P*<0.05.

## Results

### Effects of TFR on body weight of mice

**Fig 1:**
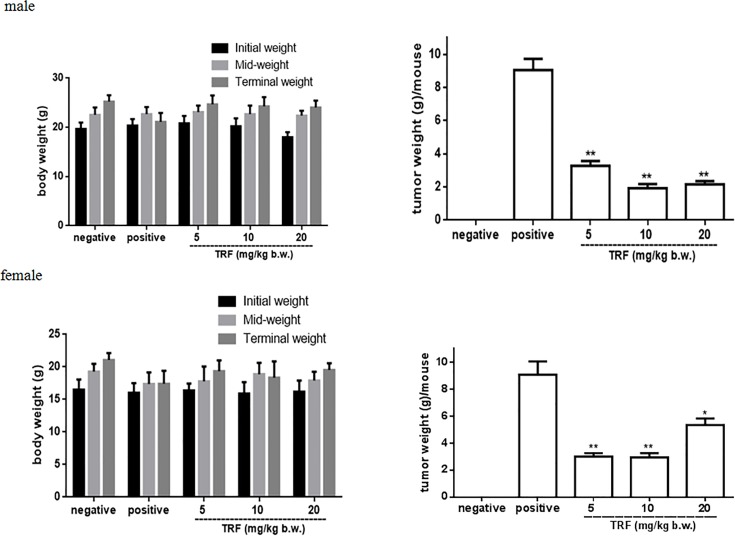
Effects of TRF on body weight and tumor weight. * p<0.05, ** p<0.01, compared to the positive control group.

As shown in Fig. 1, TRF did not affect the body weight (male and female) in the middle and terminal weight when compared to the control group (*p*>0.05). The index of body weight also showed no differences in the experimental termination between TRF treatments and control groups (**Table A in S1 File**).

### Effects of TRF on indexes of hematology and classification of leukocytes of mice

The indexes of hematology including white blood cell, red blood cell, hemoglobin, hematocrit, blood platelet and cogulation time were determined in this study (**Table B in S1 File**). The results showed that except for 20mg/kg b.w. of TRF group, TRF did not affect the indexes of hematology in the male mice. TRF at 20mg/kg b.w. group significantly decreased the number of white blood cells and increased cogulation time in comparison with the control group (*p*<0.05). In addition, the number of blood platelet in TRF and model groups showed a decreasing trend when compared to the negative control group. In female mice, TRF did not found any differences in indexes of hematology between TRF treatments and negative control groups. The classification of leukocytes also was determined in this study. As shown in **Table C in S1 File**, no differences were found in classification of leukocytes in male mice between TRF treatments and control groups (*p*>0.05). In female mice, TRF at doses of 5 or 10 or 20 mg/kg b.w. significantly increased neutrophile granulocyte or lymphocytes or eosnophils when compared to the control mice (*p* <0.05).

### Effects of TRF on the index of oxidation in the liver of mice

After given TRF for 4 weeks, the index of anti-oxidation or oxidation such as SOD, GSH-P, CAT and MDA in the livers was determined in this study. As shown in Table 2, the levels of SOD and GSH-Px did not have difference between negative and positive groups. However, the level of CAT in the positive group was significantly decreased in comparison with the negative group (*p*<0.01 or *p*<0.05). The liver antioxidant enzymes (SOD, GSH-Px and CAT) activity was increased in TRF groups when compared to the positive control group. Level of SOD also significantly increased at 20 mg/kg b.w. of TRF group in male nude mice and 5 and 10 mg/kg b.w. of TRF groups in female nude mice. GSH-Px content significant increased at 5 mg/kg b.w. of TRF group in female nude mice. CAT level was significantly increased at 5, 10 and 20 mg/kg b.w. of TRF group in male nude mice and 5 and 20 mg/kg b.w. of TRF groups in female nude mice. Level of lipid peroxide (MAD) was significantly increased at 10 and 20 mg/kg b.w. of TRF group in male nude mice and 5 mg/kg b.w. of TRF groups in female nude mice.

**Table 2:** Effects of TRF on anti-oxidative enzymes and MDA in the liver oxidation of the nude mice (n = 10, mean ± SD).

	Group (mg/kg b.w.)	SOD (U/mg•Pro)	GSH-Px (U/mg•Pro)	CAT (U/mg•Pro)	MDA (U/mg•Pro)
male	negative control	82.44 ± 8.33	54.07 ± 6.70	484.62 ± 122.81	1.07 ± 0.13
	positive control	79.79 ± 11.11	52.95 ± 6.56	211.93 ± 34.20^#^	1.44 ± 0.37^#^
	5.0	76.46 ± 9.56	51.63 ± 11.74	290.36 ± 71.20*	1.70 ± 0.28
	10.0	72.25 ± 5.34	50.94 ± 9.34	298.83 ± 41.31*	1.79 ± 0.29*
	20.0	94.38 ± 6.96*	51.88 ± 10.54	327.56 ± 47.52*	2.00 ± 0.32*
female	negative control	78.66 ± 13.28	28.02 ± 3.58	376.77 ± 28.17	1.17 ± 0.22
	positive control	84.24 ± 18.82	31.70 ± 3.85	201.30 ± 37.68^#^	1.24 ± 0.26
	5.0	98.48 ± 13.62*	38.43 ± 6.39*	388.11 ± 121.59*	2.02 ± 0.64*
	10.0	65.66 ± 8.61*	34.32 ± 1.97	257.09 ± 69.75	1.86 ± 0.65
	20.0	77.25 ± 9.29	35.44 ± 4.47	322.06 ± 53.38*	1.75 ± 1.00

SOD: Superoxide Dismutase; GSH-Px: Glutathione peroxidase; CAT: Catalase; MDA: Malondialdehyde.

* p<0.05, when compared to the positive control group.

^#^ p<0.05, when compared to the negative control group.

### Effects of TRF on tumor weight of mice

At the termination of experiment, human colon cancer xenografts in Balb/c nude mice were removed and measured the weight in this study. The results are shown in Fig. 1. TRF significantly decreased the weight of xenografts when compared to the positive control group (*p*<0.01). However, TRF at 20 mg/kg female group showed a decreasing xenografts weight. No difference was found between high dose of TRF and positive groups.

### Effects of TRF on expression of Wnt pathways related protein in tumor tissues

**Fig 2:**
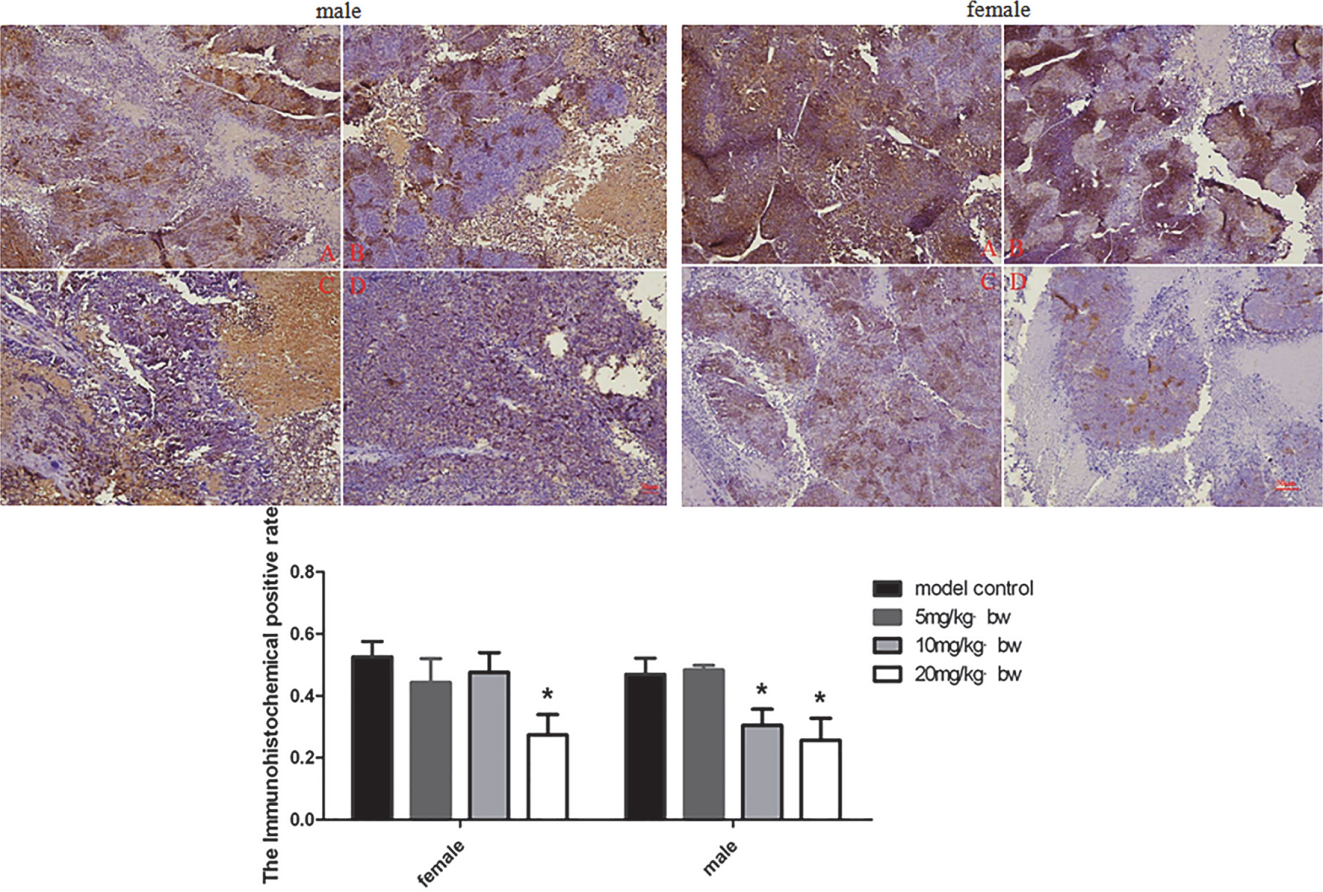
Expression of β-catenin in tumor tissues of mice treated with different doses of TRF. A, positive control group; B, 5mg/kg b.w.; C, 10 mg/kg b.w. D 20 mg/kg b.w. * p<0.05, compared to the positive control group.

**Fig 3:**
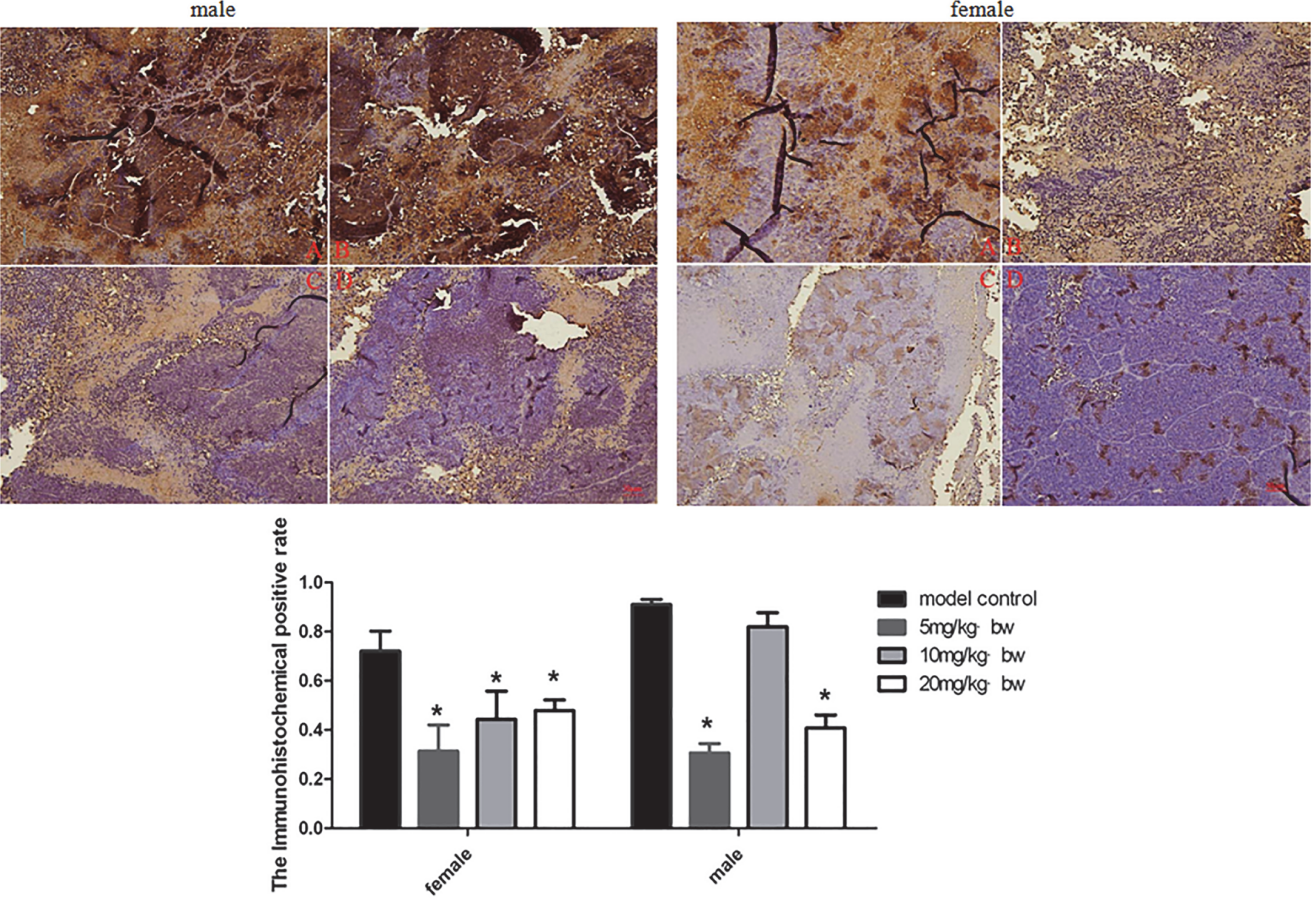
Expression of Wnt-1 in tumor tissues of mice treated with different doses of TRF. A, positive control group; B, 5mg/kg b.w.; C, 10 mg/kg b.w. D 20 mg/kg b.w. * p<0.05, compared to the positive control group.

**Fig 4:**
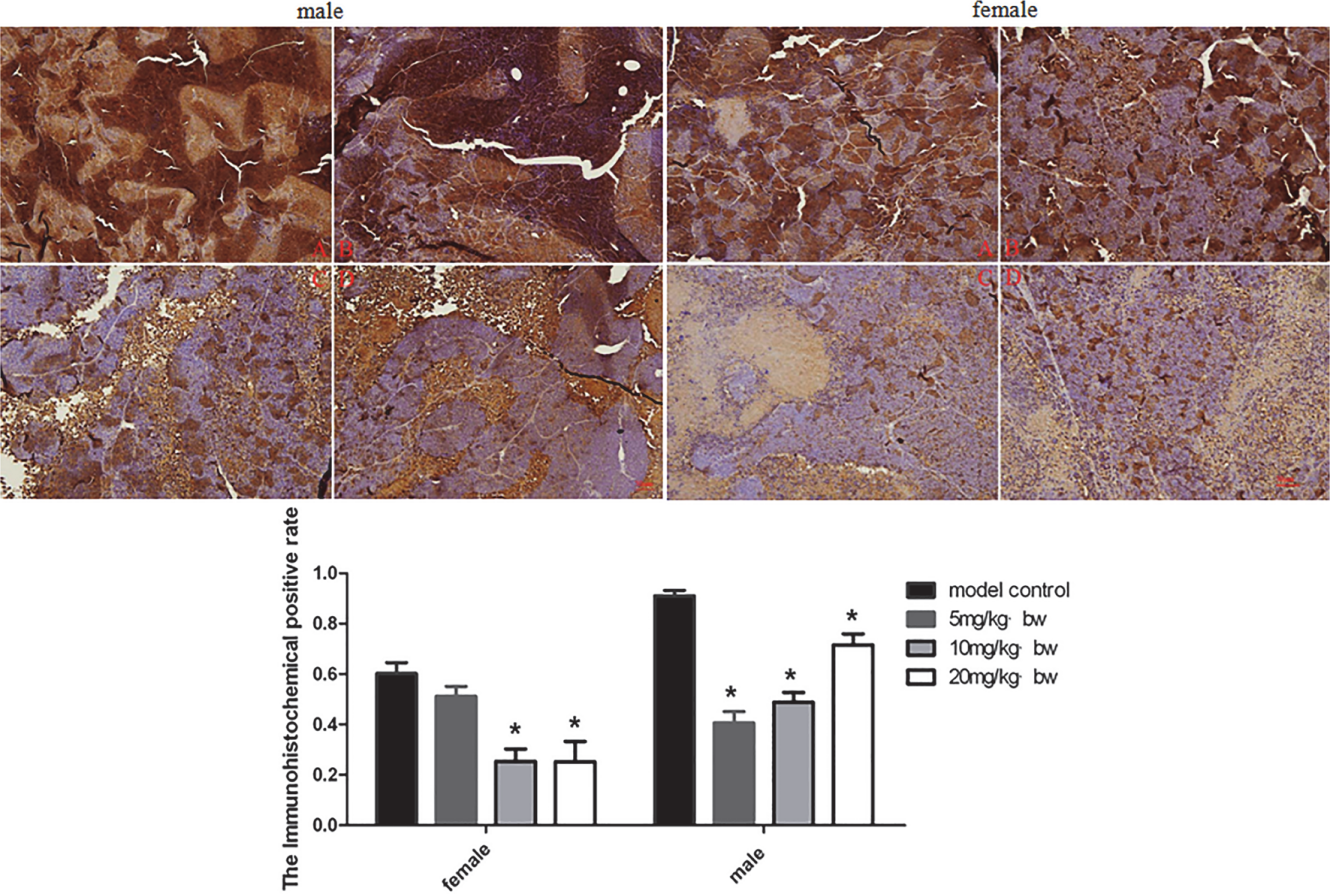
Expression of c-myc in tumor tissues of mice treated with different doses of TRF. A, positive control group; B, 5mg/kg b.w.; C, 10 mg/kg b.w. D 20 mg/kg b.w. * p<0.05, ** p<0.01, compared to the positive control group.

In order to determine the possible mechanism of TRF on inhibition of xenografts growth, the expression of Wnt pathway related protein was examined in this study. The expression of β-catenin, Wnt and c-myc in xenografts was explored by immunohistochemistry. The results are shown in Figs. 2–4. TRF significantly decreased expression of β-catenin in a dose-dependent manner (*p*<0.05) (Fig. 2). TRF also significantly decreased expression of Wnt protein in xenografts when compared to the positive control group (*p*<0.05) (Fig. 3). In addition, TRF also decreased the expression of c-myc protein in xenografts in comparion with the positice control group (*p*<0.01) (Fig. 4). Interestingly, except for female mice, c-myc expression in male mice showed an increasing trend accompanying with increasing doses of TRF.

**Fig 5:**
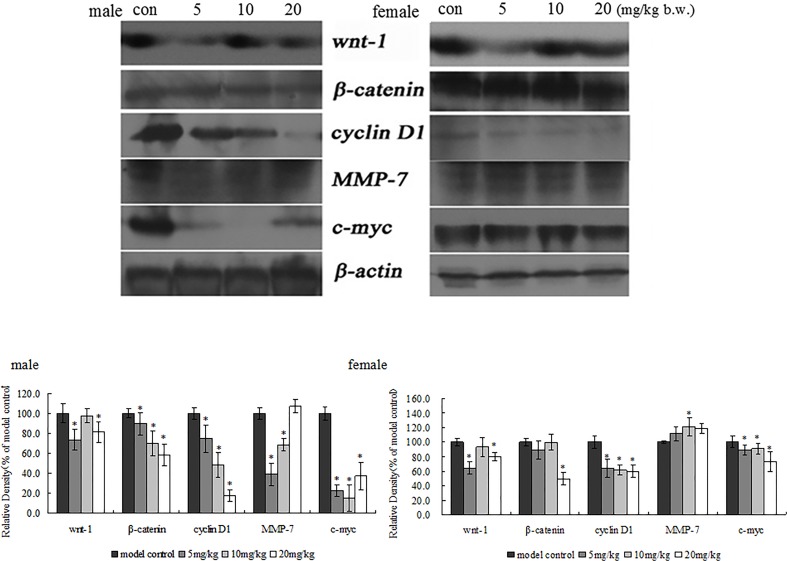
Effects of TRF on protein expression of wnt-1, β-catenin, cyclin D1, MMP-7 and c-myc in the tumor tissues of mice. * p<0.05, compared to the positive control group.

**Fig 6:**
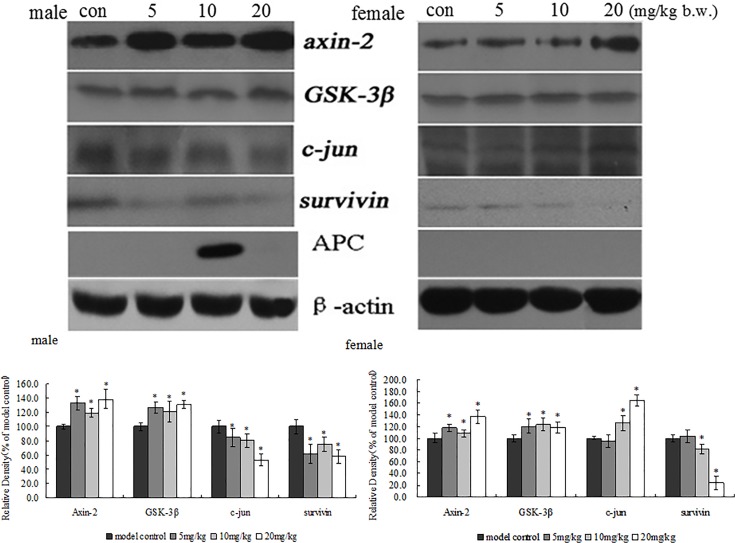
Effects of TRF on protein expression of Axin-2, GSK-3β, c-jun and surviving in the tumor tissues of mice. * p<0.05, compared to the positive control group.

In order to confirm immunohistochemical results of β-catenin, Wnt and c-myc in xenografts, β-catenin, Wnt and c-myc protein expression also was examined in xenografts by western blotting. The results are shown in Fig. 5. TRF also significantly decreased the expression of β -catenin, Wnt and c-myc protein in xenografts when compared to the positive control group (*p*<0.05). These results have the same trend as the findings with immunohistochemistry. To further determine the possible mechanisms of TRF on inhibition of xenograft growth, Wnt pathways related protein such as cyclin D1, MMP-7, axin-2, c-jun, APC, surviving, GSK-3β was determined in this study. As shown in Figs. 5 and 6, TRF significantly decreased the expression of cyclin D1 and survivin in xenografts. TRF significantly increased the expression of Axin-2 and GSK-3β in xenografts. However, TRF showed a different trend in expression of MMP-7 and c-jun in xenografts between male and female mice in comparison with the positive control group.

## Discussion

Palm oil mainly contains two kinds of common fatty acids, palmitic acid (C16) and oleic acid (C18), which have been used as a natural food over five thousand years. Palm oil not only can be easy digesting and absorb, but also is benefit to human body because of high solid glycerin which prevent the food from hydrogenating and keep it steady to be suitable for hot climate as a good seasoning to the pastry and bread. Refined palm olein has rich in tocopherols and tocotrienols, which are isomers of vitamin E. Vitamin E, a fat-soluble vitamin, is extracted from palm oil, mainly containing α-, β-, γ-, δ-tocotrienol, or α-tocopherol. Thus, palm oil is considered as a mixture of vitamin E, which is known as fractions from palm tocotrienol-rich oil (TRF). TRF has many biological activities, especially tocotrienols reduced atherosclerosis of rabbits, which may be related to the antioxidant activity of the TRF[20], improving pancreatic inflammation and fibrosis in DBTC-induced CP rats[21], preventing or treatment of diseases in dogs[22], neuroprotective effects [23], and anti-cancer activities[2]. In our research group, we used high dose of 20 mg/kg of TRF which were equivalent to 1.09 mg vitamin E/mice per day. It is lower than that in daily intake of tocotrienols (1.9–2.1 mg/day/person) for Japanese[24] and Finish diet (10.3mg α-tocotrienol/day/person)[25]. In our previous studies, tocotrienols showed an inhibiting growth of colon cancer SW620 and HT29 cells [1, 2]. Although a dose-dependent relationship of tumor weight in female mice was not see in this study, inhibition of tumor growth was observed in each group. It may be low absorption in the high dose of TRF group during a few weeks feeding. Thus, in this study, our findings showed that TRF had potential inhibition of inoculated human colon cancer SW620 cells to nude mice, including effects on tumor growth, antioxidant levels in the liver, and possible mechanisms.

### TRF enhanced activities of antioxidation in the liver

Environmentally harmful factors such as ultraviolet radiation, ionizing radiation, exposed to chemical carcinogens, etc. are exposed to organisms to produce free radicals, such as reactive oxygen radicals (ROS), reactive nitrogen species (RNS). In pathological conditions, if free radicals exceed defense capabilities of the antioxidant defense system, free radicals can be accumulated in the organisms to cause the oxidative stress. Biofilm phospholipid polyunsaturated fatty acid residues are extremely sensitive to free radicals, which is the most important target for radical attack to occur lipid peroxidation[26, 27]. Lipid peroxidation metabolites can attack DNA bases, which caused a variety of oxidative DNA damages to increase the instability of the genome. This is a common that free radicals aggregation and antioxidant function disorders always happen in cancers[28]. Tocotrienols scavenged free radicals to against lipid peroxidation. Tocotrienols exhibited a strong antioxidant capacity than tocopherols[29]. When tocotrienol and tocopherol have been given to rats or people, tocotrienol showed an oxidation protective effect, but no effect on tocopherol[30]. In our study, small amount of tocotrienols and α-tocopherol is found in the TRF. However, TRF showed decreased human colon cancer xenografts in nude mice. Antioxidative activity of tocotrienols is achieved by inducing antioxidant enzymes such as Superoxide dismutase (SOD) and catalase (CAT)[31], reduced coenzyme II (NADPH), glutathione peroxidase (GSH-Px)[32]. These enzymes quench free radicals including super oxygen free radical[33], protect and maintain the health of organism. In this study, TRF could increase the activity of CAT in mice. It suggested that SW620 cells could affect the antioxidant defense system, making mice in oxidative stress. TRF could increase the activities of SOD and GSH-Px to quench the free radicals in order to protect the health conditions. But we did not explain why TRF also increased the level of MDA which is reflected to the levels of lipid oxidation.

### TRF inhibited xenografts in mice by a regulation of Wnt pathways

Wnt pathways play an important role in several complex process of biochemical reactions in cells. In normal non-proliferating cells, such as epithelial cells of the colon, Wnt signals are weak and only a very small amount of the intracellular β-catenin protein[34]. The activation of Wnt signals in intestinal cells may induce the over proliferation of the intestinal crypt particle and eventually, lead to the occurrence of colon cancer[35, 36]. β-catenin controls the canonical pathway and is regulated by the destruction complex, consisting of two ser/thr kinases, GSK-3β (glycogen synthase kinase-3β) and CKI, two scaffold proteins, Axin and APC (adenomatous polyposis coli)[37]. When the Wnt/wg ligand is absent, Axin complex in the cytoplasm in a stable state, CK1α, GSK3β in the complex sequentially phosphorylated β-catenin, phosphorylated β-catenin in specific sites could be recognized by the E3 ubiquitin ligase enzyme β-Trcp, and then be de-gradated by proteasome, to ensure the concentrate of the β-catenin maintained at a relatively low level[38]. Once Wnt signalings are activated, Wnt protein bound with Frizzled (Fz) receptors, the activated Fz recruited Dsh to the membrane, and combined with Axin in the complex to depolymerization of the complex which concluding APC, Axin and GSK-3β[39]. The complex depolymerization and β-catenin in the cytoplasm are cumulated, then tranlocation to nuclear and combined with the TCF(T-cell factor) to activate the wnt gene which can induce the downstream genes such as c-myc, cyclin D1, survivin transcription associated with malignancy[40]. Protein phosphatase PP1 and PP2A are also involved in this process. These factors can be combined with Axin, and APC to dephosphorylate Axin and β-catenin and inhibit the complex formation and degradation of β-catenin[5]. Secreted frizzled-related proteins (SFRPs), the Wnt antagonists, as a family of new colorectal tumor suppressor candidate genes, could inhibit Wnt signaling and the expression of β-catenin target genes cyclin D1 and c-myc in β-catenin-activated colon cancer SW480 and HCT-116 cells, which lead to inhibiting the tumor growth[41, 42].

Photochemicals such as esculetin [43], luteolin[44], dioscin[45] and resveratrol [46] have been reported to anti-colon cancer through a regulation of Wnt/β-catenin pathway. Wnt signaling pathway is concerned to be a potential target for novel colon cancer therapeutics. In addition, tocotrienols have a strong anti-tumor effect and the regulation of Wnt signaling pathway which may play a role in anti-tumor mechanism. γ-Tocotrienol can inhibit the angiogenesis of SGC-7901-CM-induced HUVECs, which is of fundamental importance in tumor growth, and the inhibitory effect is related to decrease the β-catenin and cyclin D1 activity[47]. Tocotrienol also down-regulated the expression of β-catenin in cancer stem cells[48]. In our previous study, δ-, γ-Tocotrienol have shown the inhibitory growth on human colon cancer cells SW620 and HCT-8 via suppressing the activity of Wnt signaling pathways[1, 2]. In order to explore whether tocotrienols also show anti-tumor effects *in vivo*, human colon cancer xenografts in Balb/c nude mice were used to further study the similar inhibition effect of tumor growth mediated by TRF. TRF is extracted from palm oil and enriched in tocotrienols. TRF has showed the anti-cancer biologic function and also found to decrease the levels of IL-6, IL-8 and VEGF related to angiogenic in HUVEC and 4T1 mouse mammary cancer cells[49]. After supplement TRF, the expression of IL-24 mRNA, a cytokine reported to have antitumor effects, increased 2-fold in the tumor tissues of BALB/c mice[50]. Our findings showed that TRF significantly decreased the weight of xenografts, increased the expression of Wnt pathways related factors Axin-2, GSK-3β, APC and decreased the protein expression of wnt-1, β-catenin and β-catenin target genes cyclin D1, c-myc, surviving in xenografts of nude mice. These data indicated that TRF inhibited the growth of human colon cancer xenografts in nude mice by the suppression of Wnt pathways. Previous studies also showed that suppression of the Wnt signaling pathway was related to the inhibition of colon cancer cells proliferation and differentiation, induction apoptosis, suppression colonic tumor development[51, 52].

Although α-tocopherol and tocotrienols are known as a potent antioxidant and potential anti-tumor agents, the relationship between the capacity of antioxidant and antitumor is not clear. It is interesting that the Wnt signaling pathway is not only associated with tumor development, but also involved in Oxidative Stress[53, 54]. However, annatto-tocotrienol supplementation induced apoptosis and senescent-like terminal proliferation arrest in tumor cells and in HER-2/neu transgenic mice, and increased ROS production in tumor cells[55]. This indicated that the anti-tumor activity of tocotrienol might be related to the induction of oxidative stress. In our study, TRF increased the activities of GSH-Px and CAT in the liver of mice. However the exact mechanisms of TRF on inhibitory tumor growth and regulating antioxidant index needed to be further studied.

In summary, tocotrienol-rich fraction from palm oil (TRF) inhibited the growth of human colon cancer xenografts in Balb/c nude mice. This inhibition partly contributed to increase the system of antioxidative enzymes and affect the expression of Wnt pathways related to protein. Thus, TRF has potent inhibited the growth of tumor *in vivo*.

## Supporting Information

S1 File Contains Tables A, B, C. Table A in S1 File, the influence of the TRF on the organ coefficient of BALB/c nude mice. Table B in S1 File, effects of TRF on indexes of hematology in the nude mice. Table C in S1 File, effects of TRF on classification of leukocytes in the nude mice.
(DOCX)
