# Supplementary material for: Tocotrienol-Rich Fraction (TRF) Suppresses the Growth of Human Colon Cancer Xenografts in Balb/C Nude Mice by the Wnt Pathway
Source: PLoS One. 2015 Mar 25;10(3):e0122175. doi: 10.1371/journal.pone.0122175 (PMC4373919; doi:10.1371/journal.pone.0122175)
Supplement: S1 File — Table A in S1 File, the influence of the TRF on the organ coefficient of BALB/c nude mice. Table B in S1 File, effects of TRF on indexes of hematology in the nude mice. Table C in S1 File, effects of TRF on classification of leukocytes in the nude mice. (DOCX) [file pone.0122175.s001.docx]

**Supplements**

**Effects of TRF on organ weight of liver, kidney and spleen**

At termination of experiment, main organs such as liver, kidney and spleen from mice were removed and weighted. The results showed no differences in each group (p>0.05) (S1 Table).

**Table A The influence of the TRF on the organ coefficient of BALB/c nude mice (n=10)**

| Sex | Group | Liver | | | Spleen | | | Kidney | | |
| --- | --- | --- | --- | --- | --- | --- | --- | --- | --- | --- |
| Male | negative control | 5.58 | ± | 0.37 | 0.37 | ± | 0.08 | 1.38 | ± | 0.16 |
|  | model control | 5.01 | ± | 0.61 | 0.33 | ± | 0.09 | 1.31 | ± | 0.14 |
|  | 5mg/kg·bw | 5.99 | ± | 0.49 | 0.34 | ± | 0.08 | 1.37 | ± | 0.10 |
|  | 10mg/kg·bw | 5.56 | ± | 0.57 | 0.39 | ± | 0.17 | 1.32 | ± | 0.10 |
|  | 20mg/kg·bw | 5.46 | ± | 0.41 | 0.35 | ± | 0.09 | 1.36 | ± | 0.10 |
| Female | negative control | 5.31 | ± | 0.62 | 0.47 | ± | 1.15 | 1.25 | ± | 0.06 |
|  | model control | 5.21 | ± | 0.56 | 0.38 | ± | 0.13 | 1.31 | ± | 0.19 |
|  | 5mg/kg·bw | 5.58 | ± | 0.30 | 0.45 | ± | 0.08 | 1.25 | ± | 0.12 |
|  | 10mg/kg·bw | 5.43 | ± | 0.68 | 0.41 | ± | 0.10 | 1.26 | ± | 0.14 |
|  | 20mg/kg·bw | 5.40 | ± | 0.51 | 0.42 | ± | 0.13 | 1.23 | ± | 0.14 |

**Effects of TRF on** **hematology index**

At termination of experiment, plasma was obtained from mice. The white blood cell, red blood cell, HGB, HCT, platelet count, and white classification of the blood were determined in this study by an automated hematology analyzer. The results have shown in S2-3 Tables. Most of hematology indexes did not have differences between experimental and control groups. TRF significantly increased the cogulation time in 20 mg/kg b.w. when compared to the control group (p<0.05). Lymphocyte and eosinophils in the classification of leukocyte were significantly increased in 10 mg/kg b.w. of TRF in comparison with the control group (p<0.05).

**Table B** Effects of TRF on indexes of hematology in the nude mice (n=10, mean ± S.D.)

| sex | group | white blood cell (×10^9^/L) | | | red blood cell (×10^12^/L) | | | hemoglobin  (g/L) | | | hematocrit  (%) | | | blood platelet  (×10^9^/L) | | | cogulation time (s) | | |
| --- | --- | --- | --- | --- | --- | --- | --- | --- | --- | --- | --- | --- | --- | --- | --- | --- | --- | --- | --- |
| male | negative control | 7.34 | ± | 1.63 | 10.15 | ± | 0.63 | 166.62 | ± | 11.32 | 50.68 | ± | 2.86 | 712.25 | ± | 297.82 | 35.38 | ± | 4.93 |
|  | model control | 5.53 | ± | 1.44 | 10.07 | ± | 1.04 | 153.88 | ± | 16.51 | 46.83 | ± | 4.76 | 111.80 | ± | 450.44 | 43.65 | ± | 12.01 |
|  | 5mg/kg·bw | 8.34 | ± | 2.62 | 10.89 | ± | 0.57 | 169.12 | ± | 8.99 | 51.70 | ± | 2.63 | 120.85 | ± | 285.52 | 39.05 | ± | 4.91 |
|  | 10mg/kg·bw | 6.62 | ± | 1.42 | 10.38 | ± | 1.40 | 165.25 | ± | 20.02 | 50.26 | ± | 5.07 | 121.06 | ± | 251.97 | 41.23 | ± | 8.81 |
|  | 20mg/kg·bw | 4.95 | ± | 1.55* | 10.05 | ± | 1.09 | 157.38 | ± | 14.18 | 47.91 | ± | 4.42 | 113.30 | ± | 345.91 | 47.51 | ± | 10.20* |
| female | negative control | 6.81 | ± | 1.81 | 10.52 | ± | 0.68 | 172.00 | ± | 4.41 | 51.75 | ± | 2.16 | 102.08 | ± | 323.29 | 33.34 | ± | 5.75 |
|  | model control | 3.93 | ± | 1.60* | 10.55 | ± | 0.55 | 164.62 | ± | 9.74 | 49.8 | ± | 2.93 | 980.62 | ± | 227.58 | 53.63 | ± | 18.07 |
|  | 5mg/kg·bw | 5.89 | ± | 1.73 | 9.94 | ± | 0.61 | 162.88 | ± | 9.07 | 48.78 | ± | 2.85 | 899.88 | ± | 405.43 | 35.25 | ± | 6.78 |
|  | 10mg/kg·bw | 4.25 | ± | 1.38 | 10.88 | ± | 0.49 | 171.00 | ± | 6.32 | 51.65 | ± | 2.26 | 116.81 | ± | 169.25 | 43.90 | ± | 11.62 |
|  | 20mg/kg·bw | 7.62 | ± | 2.10 | 10.36 | ± | 0.98 | 161.38 | ± | 11.89 | 48.79 | ± | 4.20 | 140.71 | ± | 294.358 | 46.54 | ± | 8.55 |

* p<0.05, compared to the negative control.

**Table C** Effects of TRF on classification of leukocytes in the nude mice (n=10, mean ± S.D.)

| sex | group | classification of leukocyte | | | | | | | | | | | |
| --- | --- | --- | --- | --- | --- | --- | --- | --- | --- | --- | --- | --- | --- |
|  |  | neutrophile granulocyte | | | lymphocyte | | | monocyte | | | eosnophils | | |
| male | negative control | 58.45 | ± | 4.07 | 2.20 | ± | 2.24 | 3.79 | ± | 1.16 | 0.08 | ± | 0.21 |
|  | model control | 49.17 | ± | 11.60 | 2.54 | ± | 1.49 | 3.59 | ± | 0.93 | 0.00 | ± | 0.00 |
|  | 5mg/kg·bw | 53.33 | ± | 3.86 | 4.16 | ± | 2.20 | 3.38 | ± | 0.73 | 0.08 | ± | 0.09 |
|  | 10mg/kg·bw | 51.13 | ± | 8.43 | 4.56 | ± | 1.77 | 3.00 | ± | 0.39 | 0.08 | ± | 0.14 |
|  | 20mg/kg·bw | 45.34 | ± | 9.26 | 3.68 | ± | 2.41 | 3.20 | ± | 0.97 | 0.28 | ± | 0.05 |
| female | negative control | 33.34 | ± | 5.75 | 1.65 | ± | 1.06 | 3.83 | ± | 1.41 | 0.00 | ± | 0.00 |
|  | model control | 39.73 | ± | 17.45 | 3.15 | ± | 0.87 | 3.46 | ± | 2.68 | 0.04 | ± | 0.11 |
|  | 5mg/kg·bw | 56.24 | ± | 7.60* | 4.26 | ± | 1.82 | 4.24 | ± | 1.78 | 0.01 | ± | 0.04 |
|  | 10mg/kg·bw | 45.76 | ± | 11.06 | 5.88 | ± | 3.07* | 4.35 | ± | 1.99 | 0.11 | ± | 0.14* |
|  | 20mg/kg·bw | 46.54 | ± | 8.55 | 3.64 | ± | 1.03 | 4.30 | ± | 1.62 | 0.08 | ± | 0.18* |

* p<0.05, compared to the negative control.
